# The Impact of Breakfast Consumption or Omission on Exercise Performance and Adaptations: A Narrative Review

**DOI:** 10.3390/nu17020300

**Published:** 2025-01-15

**Authors:** Matthew T. Stratton, Shelley L. Holden, Ray Davis, Austin T. Massengale

**Affiliations:** Basic and Applied Laboratory for Dietary Interventions in Exercise and Sport, Department of Health, Kinesiology, and Sport, University of South Alabama, Mobile, AL 36688, USA; sholden@southalabama.edu (S.L.H.); jrd2421@jagmail.southalabama.edu (R.D.); atm1821@jagmail.southalabama.edu (A.T.M.)

**Keywords:** breakfast, resistance training, endurance training, fat loss, muscle gain, exercise nutrition, exercise adaptations

## Abstract

Background: Breakfast is often termed the most important meal of the day. However, its importance to acute and chronic adaptations to exercise is currently not well summarized throughout the literature. Methods: A narrative review of the experimental literature regarding breakfast consumption’s impact on acute and chronic exercise performance and alterations in body composition prior to November 2024 was conducted. To be included in this review, the selected investigations needed to include some aspect of either endurance or resistance training performance and be conducted in humans. Results: These findings suggest that breakfast consumption may benefit acute long-duration (>60 min) but not short-duration (<60 min) morning endurance exercise. Evening time trial performance was consistently inhibited following breakfast omission despite the resumption of eating midday. No or minimal impact of breakfast consumption was found when examining acute morning or afternoon resistance training or the longitudinal adaptations to either resistance or endurance training. Favorable changes in body composition were often noted following the omission of breakfast. However, this was primarily driven by the concomitant reduced kilocalorie intake. Conclusions: Consuming breakfast may aid endurance athletes regularly performing exercise lasting >60 min in length. However, the morning meal’s impact on resistance training and changes in body composition appears to be minimal. Although, as the body of literature is limited, future investigations are needed to truly ascertain the dietary practice’s impact.

## 1. Introduction

The relationship between exercise, nutrition, and metabolism is complex and remains an important topic among sports scientists and athletes alike. Further, the importance of breakfast and its effects on athletic performance has often been emphasized. Its origins as the first meal of the day—the meal causing someone to break their overnight fast or “break-fast”—has led to many ambiguous definitions. For instance, some argue if one eats anytime throughout the day it is impossible to skip breakfast because they are always breaking their fast. Within the literature, breakfast is defined as consuming >50 kilocalories (kcals) within the first 2 h of waking [1,2] and as such will be our definition throughout this review. This meal is colloquially known as “the most important meal of the day” due to previous associations made between an increased frequency of breakfast consumption and improvements in health markers such as decreased body mass index (BMI) [3,4], along with reductions in the incidences of chronic diseases such as diabetes [5] and coronary heart disease [6]. However, with the rise in popularity of certain specialty diets like intermittent fasting and its most popular variant commonly referred to as timed restricted eating (TRE), which involves restricting all kilocalorie consumption to a daily <10 h period [7], intentional breakfast omission has become increasingly commonplace in Western society [8]. Previous reports suggest that as many as 30% of young people skip breakfast every day, and up to 60% eat breakfast infrequently [9,10]. Additionally, a 2024 investigation by Raleigh and colleagues found that 38% of the elite endurance athletes surveyed reported purposefully omitting the morning meal prior to morning training on a consistent basis due to their perception that training fasted would either improve body composition or metabolic adaptations to performance [11]. This occurs despite the often cited arguments for the importance of consuming breakfast for athletes including blood glucose regulation, energy for performance, improved cognitive functioning, and muscle repair [2,12,13,14,15]. Indeed, previous investigations have shown decrements in glucose regulation and insulin sensitivity following 2 weeks of breakfast (whole grain cereal) omission [16] and 11 weeks of reducing meals to a single daily 4 h period [15] in lean individuals (BMI < 25 kg/m^2^) and individuals with type 2 diabetes [17] but not in those with obesity but otherwise healthy [18,19], consuming self-selected items with shorter fasting periods (until noon) [20,21], when combined with exercise [22], or in calorie-restricted states [23]. Similarly, the reduced number of daily protein feedings, and subsequently protein synthetic stimulations, associated with skipping breakfast has been theorized to impair skeletal muscle performance and adaptations [13], which may negatively impact athletic performance. Lastly, a review by Galioto et al. [24] reported that breakfast consumption was associated with improved memory performance, but not attention or motor and executive function, in children and adults. Still, how these may impact exercise performance and adaptations is unknown.

While the majority of the focus has been placed on the impact of breakfast consumption on morning daily habits, what is often overlooked is the impact of consuming or omitting the morning meal on exercise performance that occurs later in the day (e.g., afternoon and evening) following the resumption of eating. An investigation by Buman et al. suggested that ~20% of the United States population exercises within 4 h of bedtime [25]. These data combined with the previously discussed rates of overall breakfast skipping, in athletes and the general population, suggest that a significant portion of individuals may perform afternoon or evening exercise without having consumed breakfast. To the authors’ knowledge, a summation of the current body of literature regarding how breakfast consumption may impact not only acute exercise performance but longitudinal adaptations to said exercise is lacking. Thus, the purpose of this narrative review is to summarize the available literature on how consuming or omitting breakfast may impact acute resistance or endurance training performance and chronic adaptations to endurance or muscular performance along with alterations in body composition. Additionally, this narrative review seeks to examine how breakfast consumption may play a role if said exercise is undertaken either in the morning under fasted conditions or in the afternoon and evening following the resumption of eating.

## 2. Literature Search

Literature searches were conducted by all authors using PubMed, Google Scholar, MedLine, Scopus, and the university library’s OneSearch resource using the following terms: “Breakfast” AND “Exercise” OR “Resistance Training” OR “Resistance Exercise” OR “Endurance Exercise” OR “Endurance Training” OR “Body Composition”. Additionally, since many intermittent fasting protocols ask the practitioner to omit breakfast, the above search was repeated with “Fasting” or “Time Restricted Eating” in place of breakfast. The reference and “cited by” sections of relevant articles were also scanned. To be included in this narrative review articles needed to meet the following criteria: (1) published prior to November 2024; (2) an experimental intervention conducted in humans; (3) a comparison to some form of morning breakfast consumption; (4) include an exercise component; (5) report alterations in variable(s) related to acute or chronic adaptations in response to resistance or endurance training; (6) published in the English language.

## 3. Acute Impacts on Exercise Performance

### 3.1. Endurance Exercise Performance

Nutritional strategies [26,27], optimal training [28], and recovery [29,30] are factors that contribute to high levels of performance in endurance activities. Endurance athletes are typically involved in exercise with continuous movements of 20 min up to >4 h for ultra-endurance athletes [31]. The continuous movement is usually over long distances or longer periods of time and because of the duration and continuous movement, endurance athletes, as a population, expend an exorbitant number of kilocalories during training and competition. Further, it has been determined that early fatigue in this population most often occurs from a depletion of carbohydrate stores or dehydration [32]. This is a particular note due to the popularity of either pre-exercise carbohydrate restriction or performing exercise sessions fasted. A recent investigation by Raleigh et al. in which 327 elite endurance athletes (≥8 h of training per week and competed at least at the national level) were surveyed about their dietary and exercise habits found that 38% reported performing fasted training, prior to breakfast, in an attempt to improve either body composition or metabolic adaptations [11]. Thus, an investigation into the impacts of breakfast consumption on acute endurance performance is warranted.

A 2018 systematic review and meta-analysis by Aird et al. [33] examined 37 investigations and concluded that pre-exercise morning feeding enhanced prolonged (>60 min) but not short-duration (<60 min) aerobic performance. Interestingly, exercise intensity (e.g., moderate continuous intensity vs. HIIT) did not seem to affect the influence of fed/fasted state on performance. However, the four surveyed studies that included the pre-exercise meal 3–4 h prior to the exercise session all noted enhanced performance over the fasted condition [34,35,36,37]. This could be attributed to a rise in liver glycogen stores, which may be depleted after an overnight fast [7] and might not be fully replenished if the pre-exercise meal is eaten within the 60 min before exercise [38]. However, most morning exercise situations often do not realistically allow for a meal to be consumed 3–4 h prior without disrupting the athlete’s sleep (e.g., 3 a.m. feeding prior to a 6 a.m. workout). Regular sleep disturbances may have larger unintended consequences on long-term improvements in performance. Still, these data suggest that if the planned exercise session is >60 min, pre-exercise feeding may be beneficial.

Breakfast omission has been shown to impair endurance exercise performed later in the day despite midday ad libitum food consumption [39,40,41]. Cornford et al. [39] demonstrated a small but statistically significant reduction in evening (4:30–6 p.m.) 2000 m time trial performance in a group of 10 collegiate rowers following extending the overnight fast until noon when compared to consuming an individualized (25% of estimated daily needs) carbohydrate-rich (62% CHO, 17% PRO, and 21% fat) breakfast at 9 a.m. Similarly, Clayton et al. [40] noted an ~4% reduction in 60 min evening (5 p.m. ~9 h after breakfast) cycling performance consisting of 30 min at 60% of VO_2_peak followed by a 30 min time trial in which they were instructed to complete as much work as possible following the omission of the same breakfast utilized by Cornford et al. [39], resulting in at least 14.5 h of fasting prior to the noon meal. However, in both of these investigations, participants consumed significantly less food prior to the evening exercise test (Cornford et al.: −353 kcals, −80 g CHO, −28 g PRO, +9 g fat; Clayton et al.: −654 kcals, −126 g CHO, −17 g PRO, −7 g fat) due to not fully recompensating the energy not consumed during breakfast during the ad libitum feeding periods later in the day. This suggests the differences in nutritional intake may have influenced the findings in addition to alterations in meal frequency. Nonetheless, similar to the previous findings, the lone study to control for pre-exercise energy intake still detected decrements in evening (5:00–7:00 p.m.) 20 km cycling time trial performance when participants did not consume the carbohydrate-rich breakfast (~589 kcals; 81% CHO, 13% PRO, 6% fat; 8–9 a.m.) and consumed the same total daily intake at lunch (~1457 kcals; 81% CHO, 13% PRO, 6% fat; 12–2 p.m.) [41]. However, the magnitude of performance reductions in the three previous trials calls into question their practical relevance. The decreases in performance ranged from 0.8% to 4.5%, which translated to a 3.5 s (0.8%) change in rowing time for Cornford et al. [39] and a 38 s (3.5%) increase in 20 km cycling time for Metcalfe and colleagues [41]. The ~3.5 s (~0.8%) reduction in 2000 m time trial performance seen by Cornford and colleagues [39] may be relevant for a high-level athlete at a competition but not for the recreational trainee in a regular training session. Thus, these results should be seen as highly contextual. Additionally, the interventions included are not representative of a normal training session. Most athletic workouts are going to be somewhat submaximal in nature. The designs employed in these studies [39,40,41] are more representative of a competition setting. Thus, breakfast consumption may be more imperative on competition days than throughout a training cycle. However, direct interventions are needed examining submaximal training more indicative of a typical workout and any long-term impacts. An additional consideration that needs to be made is how these energy intakes may reflect the necessary energy needs of the exercise bout itself. As energy expenditure throughout the session was not reported in these investigations, its impact is unknown. Future investigations should seek to elucidate the impact of breakfast consumption on varying styles or workouts with differing energy needs.

### 3.2. Resistance Exercise Performance

The need to consume breakfast prior to morning resistance training has been a hotly debated topic. This is largely due to the prevalence of those who regularly exercise during the early morning hours, for instance, those who prefer to exercise before work, morning PT for many military and tactical populations, and weight training sessions for collegiate athletes. Until recently, little was known as to the impact consuming breakfast may have on these acute resistance training sessions. The first study to explore this relationship was conducted by Naharudin and colleagues in 2019 in which 16 resistance-trained (resistance train at least two times per week for 2 years) males that habitually consumed breakfast performed four sets of barbell back squat and barbell bench press to momentary concentric failure with 90% of their previously established 10RM after either consuming breakfast, or only water [42]. When fasted (>12 h; average length of fast was not reported), participants completed 15% and 6% fewer repetitions on barbell back squat and barbell bench press, respectively, during the morning exercise session than after consuming a breakfast providing 1.5 g/kg of CHO and ~20% of daily energy needs (exact kcal and macronutrient breakdown was not reported) 2 h prior to exercise (~8:00–9:00 a.m.). Interestingly, the majority of the decrements in repetition performance were observed during the first two sets, and no statistical differences were noted on sets 3 and 4. These preliminary data suggest that resistance training volume early in the exercise session may be partially compromised when performed in a fasted state (e.g., skipping breakfast), although these decrements may be limited to the first few sets of a given exercise. This group performed a similar follow-up investigation with the same morning and peri workout nutrition timings in which a sham breakfast (26 kcal, 2.4 g CHO, 0.8 g PRO, 0.6 g fat) consisting of 15% low-energy orange squash and water matched for taste and texture to a carbohydrate mixture with an additional 1.5 g/kg of maltodextrin (496 kcals, 119 g CHO, 0.8 g PRO, 0.6 g fat) was included. Similar results were found to the 2019 investigation with regards to the standardized carbohydrate breakfast compared to the water conditions; however, when participants underwent the sham breakfast condition, no differences in performance were seen compared to the carbohydrate breakfast. These data suggest that some of breakfast’s impacts on morning fasted exercise performance may be psychological in nature. This will be explored in greater detail in the next section.

While the work of Naharudin and colleagues [42,43] provides valuable insight into the impact of breakfast consumption on acute morning resistance training performance, it does little to address whether consuming breakfast may impact subsequent performance after feeding is resumed (e.g., afternoon or evening performance). Our group explored this question in a group of 39 resistance-trained males (n = 20) and females (n = 19) who either habitual consumed (n = 19) or skipped (n = 20) breakfast [44]. In this investigation, following either consuming the same total amount of food at either breakfast (~11.2 h fast; 416 kcals, 67 g CHO, 17 g PRO, 9 g fat) and lunch (639 kcals, 88 g CHO, 30 g PRO, 24 g fat) or solely at lunch (~14.7 h fast; 1055 kcals, 155 g CHO, 47 g PRO, 33 g fat), participants undertook an afternoon resistance training workout (1–6 p.m., ~2 h after the lunch meal; avg start time 2:10 p.m.) comprised of four sets of barbell back squat, barbell bench press, and conventional barbell deadlift at 80% of their previously established 1RM, with the first three sets being taken until participants felt they were one repetition away from momentary concentric failure (1-RIR) and the final set being taken to momentary concentric failure. Additionally, all food was provided, and participants were given instructions as to what food they should consume when. Thus, the only difference was in the number of meals the food was divided into, not the total amount of food that was consumed, although it must be mentioned that the meals were not directly supervised, so the degree of compliance was based on verbal interviews at the start of the visit. No differences in performance were found in response to the different meal patterns. This led the researchers to conclude that when resistance training is undertaken in the afternoon, the total pre-exercise food consumption is of a greater importance than meal frequency. Therefore, provided total pre-exercise nutrition remains consistent, the consumption of breakfast does not appear to significantly impact afternoon resistance training sessions of this nature. However, as the evidence is limited, more investigations are needed using more exercise variations (e.g., high rep ranges, circuit training, high-intensity functional training, etc.) as to see if these findings extend to more than lower (<8)-repetition, high-load multi-joint full-body strength training.

### 3.3. Psychological Impacts

Recent investigations have brought into question whether the performance decrements previously noted may be mediated by more than a physiological response to fasting [43,45]. In two instances, ingestion of a placebo was shown to mitigate the performance decrements in cycling [45] and resistance training [43] performance despite the lack of energy and nutrient provision. In a 2020 investigation, Naharudin et al. [43] replicated their previously discussed study [42] but included a condition with a placebo breakfast, which the participants were told contained carbohydrates. No differences were noted between carbohydrate and placebo conditions for total repetitions in the barbell back squat. Yet, much like the 2019 study [42], back squat repetitions were still lowered in the water-only group. However, in contrast to the earlier investigation, no differences were noted for any condition for the barbell bench press. Parallel results were seen following the consumption of a similar beverage before cycling time trial performance [45]. Mears and colleagues asked 13 well-trained cyclists to consume either 7 mL/kg of water, a placebo breakfast drink (6 mL/kg water and 1 mL/kg orange squash), or a carbohydrate drink consisting of the placebo drink with an additional 2 g/kg of maltodextrin matched for taste and texture with the placebo; total energy and macronutrient intake was not reported. At the time of 90 min following ingesting the selected drink, participants underwent a 30 min exercise session that included cycling for the first 10 min at 60% of their maximum power output followed by a 376-kilojoule time trial. Equal performance was seen in the two breakfast conditions while the water condition took significantly longer to complete the time trial (water: 1146 s; placebo: 1112 s; CHO: 1120 s). These findings led the authors to suggest that for exercise modalities and durations where carbohydrate plays a lesser role, decrements in fasted performance may be more psychological than physiological [45]. Indeed, the placebo effect has been demonstrated in endurance exercise lasting up to 60 min [46]. Conversely, longer-duration exercise failed to benefit from placebo consumption [47].

The previous findings agree with the analysis of Aird et al. [33], in which exercising while fasted only impaired exercise > 60 min in length. These data point to the placebo effect playing a larger role in exercise modalities in which glycogen levels are not a limiting factor. One of the main aims of a carbohydrate meal after an overnight fast is to replenish liver glycogen [48] as muscle glycogen is minimally affected by fasting [49,50,51]. Furthermore, low-volume resistance training does not appear to greatly affect muscle glycogen levels. Reductions in muscle glycogen of up to 40% have been reported [52,53,54], with higher repetition volume needed to reach such depletion [55]. Yet, the study by Naharudin et al. [43] only included performance of eight total sets, with average repetitions falling below 10. Carbohydrate ingestion seems to exert a greater effect when multiple sets of >10 repetitions are performed to momentary muscular failure with minimal rest between sets [56], with little effect of carbohydrate being seen on sets lasting less than 10 repetitions [57]. Importantly, these works did not differentiate between habitual breakfast consumption habits. Therefore, it is also possible that the placebo effect may be present in those that habitually eat before exercise. Secondly, in both interventions [43,45] participants were asked not to perform any exercise for 48 h prior to the exercise bout, likely resulting in participants entering the session with high levels of muscle glycogen. Had the participants performed strenuous exercise to lower resting levels of muscle glycogen, the effect of carbohydrate ingestion may have been apparent due to lower levels of muscle glycogen being related to decreased performance [58,59]. However, more investigations are needed to either confirm or refute these hypotheses.

## 4. Impacts on Longitudinal Adaptations to Exercise

### 4.1. Body Mass and Composition

A significant contributor to breakfast earning the moniker of “the most important meal of the day” is due to previous investigations showing the meal’s relationship with decreased BMI [3,4]. However, it must be stated that these notions are primarily based on cross-sectional correlation-based studies [3,5,6,60]. These findings should be approached with caution as regular breakfast consumers tend to also practice healthier behaviors such as increased physical activity, consuming a higher-quality and more micronutrient-dense diet, and not smoking [61,62,63,64]. As such, reviews of direct interventional studies regarding breakfast intake on measures of weight management have been inconclusive [9,65]. Furthermore, few investigations have been conducted directly examining the impact of breakfast consumption or omission on longitudinal changes in body composition when combined with exercise. To date, the majority of those conducted have been utilizing variants of IF, most notably, TRE [22,66,67,68,69,70] or the religious practice of Ramadan [71,72]. While an expansive examination of TRE is beyond the scope of this review and has been thoroughly reviewed elsewhere regarding its impact on changes in body mass [73,74], metabolic health [75], cardiometabolic health [76,77], disease status [78,79,80,81], cognitive function [82], and aging [78], a brief overview is warranted. Intermittent fasting’s most common variant, TRE, involves restricting calorie consumption to a daily ≤ 10 h period, often later in the day (e.g., noon–8 p.m.) [7]. TRE, along with other common IF variants such as periodic fasting and alternate-day fasting, have grown in popularity throughout the literature due to previous investigations showing clinically significant improvements in body weight [83,84], glucose homeostasis [73,79,85,86], and cardiometabolic health markers [76] even independent of weight loss in some instances [18]. This is mainly because the calories missed at breakfast are not made up for during the rest of the day. Indeed, previous investigations have found that, in uncontrolled conditions, only the first meal following the resumption of eating is increased [87]. This often results in an ~20% reduction in total daily energy intake [88] or ~550–650 kcals/day [19] and 3–8% weight loss after 8–12 weeks [74]. However, it is worth noting that in ad libitum conditions, those following TRE tend to self-select the reduced energy intake to come from a reduction in protein and a greater desire for higher-fat meals [69,87]. This has led many to question the feasibility of the dietary protocol for long-term improvements in health status, muscle function, and the ability to perform activities of daily living [89]. Indeed, some investigations have shown that a portion of the reduction in weight may come from reductions in skeletal muscle mass [90]. However, it must be noted that this has only been seen in ad libitum conditions in which an inadequate amount of high-quality protein is consumed (<1.0 g/kg) and when not combined with exercise, particularly resistance training, and as such may not be representative of active athletic populations. However, this demonstrates the need to be conscious of the limitations for TRE (increased protein consumption and the inclusion of exercise) for clinical populations. Furthermore, the true implications of such dietary practices on various clinical populations (e.g., type 1 diabetics) is unknown, and as such should be approached with caution.

In healthy active populations, investigations continuously suggest that the consumption of breakfast does not impact alterations in skeletal muscle mass within 4–8 weeks. This is likely due to the fasting periods involved in TRE not impacting daily myofibular protein synthesis rates in healthy adults [91]. However, there are discrepant reports on changes in fat mass. The investigations by Tinsley [67,69] and Moro [22,68] reported those in the TRE groups (i.e., the skipping-breakfast group) lost fat mass, which was either not seen or seen to a lesser extent in the control groups (i.e., consumed breakfast). This is in contrast to the findings of our group [66] in which no differences in changes in body composition were noted regardless of the dietary protocol. These differences in findings are likely to be due to the goal of the investigations and the subsequent methodology in how they were conducted. Firstly, our investigation [66] was only 4 weeks in length while those of Tinsley [67,69] and Moro [22] were 8 weeks. This may suggest that if variations do occur, they may only be apparent in longer-duration (>4 weeks) settings. Secondly, our study was the only investigation in which the goal was true body mass loss. The two investigations by Moro [22,68] were undertaken trying to keep participants in a caloric maintenance (not gaining or losing body mass). This resulted in significantly higher prescribed dietary intakes (Moro 2016: ND: ~2900 kcals/day vs. TRE: ~2700 kcals/day; Moro 2020: ~4800 kcals/day for both conditions) over the 3–4 meals in the given 8 h eating widow, which brings the feasibility of the investigation into question. TRE often results in a reduced daily kilocalorie intake by making it more difficult for practitioners to eat as much per sitting [92]. Thus, it is likely that while these higher intakes were reported by the participants, they may not have been followed, similar to an investigation by Stote and colleagues [92] in which despite participants being provided meals and consumption being supervised, when participants were following a TRE protocol, they still consumed 65 kcals less per day of the provided 2396 kcals/day, significantly lower than the amount consumed in either Moro investigation. Furthermore, the participants in the Stote and colleagues investigation stated that had they not been supervised, they would have consumed even less due to increased feelings of fullness [92]. The studies by Tinsley and colleagues [67,69] had less dietary control which also resulted in differences in energy intake which may have been the primary driver of the differences in fat mass. The 2019 investigation [67] was undertaken with the goal of body recomposition (loss of fat mass and gain of skeletal muscle mass). As such, participants were prescribed a small deficit (~250 kcals/day) and as long as they did not report eating more than the prescribed amount during check-ins they were seen as compliant. This led to the TRE groups consuming ~132 kcals less per day than their breakfast-consuming counterparts. Similarly, the 2017 investigation by Tinsley and colleagues [69] employed a free living model in which participants were not given any dietary guidelines aside from what time windows to consume their meals. This resulted in an average of ~650 kcals less consumed per day on the four fasting days throughout the week. This is all in contrast to our 2020 investigation [66] in which participants were placed in an estimated ~25% kilocalorie deficit and had the same reported dietary intake (TRE: 1946 kcals/day vs. ND: 1936 kcals/day) throughout the four-week investigation. No differences in body composition were noted. This suggests that changes in calorie intake, not breakfast intake, are the primary mechanism underlying changes in body composition.

The omission of breakfast seen with many IF variants is frequently cited as the underlying cause for reductions in the ad libitum total daily energy intake associated with IF [7]. This phenomenon occurs due to increased energy consumption only occurring at the first uncontrolled meal of the day [93,94,95], which often fails to compensate for the omitted morning intake [39,40,95,96]. A 2020 investigation by Zeballos et al. [95] noted a 253 kcal reduction in total daily energy intake following breakfast omission. However, despite reductions in overall energy intake, many investigations have reported smaller-than-expected fat and body mass losses [97,98]. These findings are believed to be the result of reduced morning non-exercise physical activity following morning food omission [96,99]. Such compensatory behaviors have been seen in individuals with [99] and without obesity [96], although compensatory behaviors related to energy intake often appear to be more pronounced in individuals with obesity [100] while lean individuals have been reported to decrease physical activity to a greater extent (442 kcal/day vs. 272 kcal/day) [96,99]. The primary reductions arise from spontaneous light-intensity behaviors as opposed to conscious decisions not to participate in structured physical activity or exercise. As such, these subconscious alterations in activity may not be detected using devices such as physical activity questionnaires. However, the divergence of compensatory mechanisms based on weight status may be in part due to individuals with obesity typically displaying lower baseline physical activity levels. Therefore, a similar relative reduction would result in a smaller absolute reduction in physical activity energy expenditure.

The timing of exercise in conjunction with breakfast has demonstrated an ability to affect 24 h energy balance as well. More specifically, previous investigations have examined the impact of morning exercise with or without breakfast consumption on 24 h energy balance [101,102,103]. All three prior investigations found that morning moderate-intensity (50–60% VO_2_max) endurance exercise, in conjunction with breakfast omission, resulted in a negative energy balance over a 24 h period. Interestingly, Bachman et al. [101] was the sole investigation to report energy expenditure during the exercise bout. The breakfast omission condition resulted in the greatest total daily energy deficit despite approximately 4% lower energy expenditure throughout the exercise session. Furthermore, the exercise respiratory quotient was lowered in the breakfast omission group (0.90 versus 0.86). However, the average relative VO_2_max in this investigation was 59.1 mlO_2_/kg/min, and the mean VO_2_max across all three studies was 55 mL of O_2_/kg/min (53, 59.1, and 53.1) [101,102,103], indicating that these findings might not extend to lesser-trained or clinical populations.

Lastly, the role of habitual breakfast consumption must not be overlooked. Few studies have directly compared habitual breakfast consumers and skippers [65]. The majority of examinations have utilized cohorts of purely habitual consumers (≥5 days/week) [1,16,104,105,106,107] or skippers (≤2 days/week) [9,108,109,110,111]. Thus, direct comparisons between the two behavioral patterns remain difficult. Future investigations should examine mixed cohorts to better ascertain the impact of habitual breakfast patterns and time courses for potential adaptations, if any. Furthermore, if the goal of the individual is to improve body composition, despite either consuming or omitting breakfast, the primary emphasis should be placed on total nutrient intake and be cognizant of the potential to decrease dietary quality whenever meal frequency is reduced. These data also suggest that skipping breakfast would not be recommended in athletes or individuals who either need to increase body mass (e.g., NFL lineman or various clinical populations) or have a difficult time maintaining their necessary kilocalorie intake for health reasons or their chosen sport.

### 4.2. Muscular Performance

The improvement or maintenance of muscular strength and endurance performance is a common concern amongst athletes from recreational to advanced levels. Whether due to the demands of daily life, dietary preference, or religious belief, breakfast is often skipped, which opens the question of how breakfast impacts longitudinal adaptations in muscular performance in response to training interventions. Unfortunately, the literature on this topic is sparse; however, investigations into fasting—both religious (e.g., Ramadan) and intermittent (e.g., TRE)—may give more insight as the daily fast (>14) [7,112] can mimic the timeframe seen by those skipping breakfast. Triki and colleagues examined the impact of performing whole-body resistance training 4 days per week either fasted in the late afternoon or fed in the evening following the first meal after the sun had set [72]. The researchers noted significant improvements in squat and deadlift 1RM in the group training after evening food consumption while no differences were found (positive or negative) in the fasted training group from pre to post timepoints. Rebaï et al. sought to examine the impact of resistance training volume during fasted training throughout Ramadan [113]. Following a 4-week preparatory resistance training program, 20 offseason male soccer players were randomized into either a group that trained at the same resistance training volume utilized during the preparatory phase or a tapered group in which the total training volume decreased by 22% per session resulting in 48% per week while intensity remained the same. Muscular performance was assessed via squat and countermovement jumps, along with maximal voluntary isometric contractions (MVICs) of the knee extensors. Following the 30 days of Ramadan, the normal training group experienced decreases in performance or maintenance while the tapered group improved performance throughout the length of the investigation. These findings suggest that those training fasted (e.g., having not consumed breakfast beforehand) may benefit from a reduced session volume and could be at a higher risk for overtraining leading to suppressed adaptations. These findings are not without limitations as previous investigations have determined that Ramadan fasting is unique compared to traditional intermittent fasting regimens due to the changes in total energy intake/expenditure along with psychological changes. Findings from Osman et al. (2020) and Lessan et al. (2018) found that during Ramadan fasting, participants are more likely to have a lower daily caloric intake and have a reduction in daily energy expenditure [114,115]. Additionally, these aspects depend on the type of fast conducted. During Ramadan, fluid consumption is restricted as well, so dehydration becomes a greater concern that may not be apparent during a typical overnight or TRE-style fast. For the impact of breakfast consumption on muscular adaptations when training is performed later in the day in a fed state, the available TRE literature can be beneficial.

Unlike the divergent findings regarding TRE and alterations on body composition, the respective findings on performance adaptations are relatively consistent. Improvements in upper-body and lower-body maximal strength have been seen in investigations lasting 4–8 weeks [22,66,67], similarly with lower-body metrics on muscle power like the vertical jump [66]. However, findings regarding improvements in muscular endurance are mixed. Tinsley et al. noted improvements in repetitions to failure in adult females following 8 weeks of TRE [67]. Conversely, we failed to detect any improvements in repetition performance during the same test following 4 weeks of TRE in adult males [66]. The differences in findings likely stem from the method chosen in which to assess changes in muscular endurance performance. Tinsley and colleagues [67] had participants perform repetitions to failure with 70% of their baseline 1RM at pre- and post timepoints, while we had participants utilize 65% of their 1RM and adjusted for their new 1RM at the end of the 4 weeks [66]. Therefore, as both investigations noted improvements in 1RM, the lack of improvements in repetitions to failure likely stem from the participants in our investigation using a higher weight at post, while those in Tinsley et al.’s study did not, thus obtaining a lower relative intensity (%1RM). Finally, an important factor to note is that while the TRE groups in the described investigations all omitted breakfast on a daily basis, all training was conducted in a fed state in the afternoon. Therefore, these data do little to elucidate the impact of breakfast consumption if the resistance training is performed in the morning under either fed or fasted conditions. Future investigations examining breakfast consumption directly—not TRE—are needed in order to provide a clear consensus as to its impact on longitudinal adaptations in muscular performance.

### 4.3. Endurance Performance

Improving endurance exercise performance is essential, as it contributes to enhanced cardiovascular health, greater physical stamina, and the ability to sustain prolonged physical activity, all of which are fundamental for both athletic success and overall well-being. Furthermore, the majority of exercise recommendations are based around performing cardiovascular endurance exercise. It has been well established that dietary manipulation, for instance, the carbohydrate or fat content of one’s diet, can impact acute endurance performance. However, little attention has been paid to long-term dietary practices and their impact on endurance exercise performance adaptations, especially in regard to the role of breakfast consumption. Although, some common dietary practices among endurance athletes involve either selectively eliminating or delaying the first meal of the day in order to perform fasted exercise sessions (e.g., carbohydrate periodization), or eliminate the morning meal all together (e.g., TRE). While an in-depth overview of carbohydrate periodization is beyond the scope of this review (for a greater exploration please refer to the reviews by Hawley et al. [116] and Bertlett et al. [117]), a basic understanding is warranted. Those employing many carbohydrate periodization protocols often manipulate carbohydrate intake prior to exercise sessions in order to commence the bout either in a high- or low-carbohydrate state [116]. For instance, one might perform a high-volume bout of endurance exercise in the afternoon/evening, then refrain from consuming carbohydrates until after a fasted training session in the morning. Previous reports have suggested these techniques may enhance cellular adaptations to endurance exercise like mitochondrial density [116,117]. However, longitudinal evidence is mixed as to whether these practices result in increased exercise performance [118,119]. A 2016 investigation by Marquet et al. [119] noted significant improvements in submaximal cycling efficiency and 10 km running performance when triathletes underwent 3 weeks of carbohydrate periodization which included performing some sessions fasted prior to the morning meal compared to controls who consumed the same daily amount of carbohydrates (6 g/kg/day) spread equally throughout the day. However, Burke and colleagues failed to detect similar improvements in Olympic-caliber race walkers after 3 weeks of a similar dietary protocol [118], although neither investigation stated the length of time of the overnight fast along with the length of time between finishing the exercise session and the resumption of eating, which are both important considerations and can potentially impact outcomes. It is also important to note that these fasted sessions are often limited to predetermined low-intensity bouts due to likely decreases in self-selected pace and intensity [116], as well as the potential inability to maintain higher training intensities [117] during fasted sessions. Thus, the impact of habitually performing all of one’s training sessions in the morning under fasted conditions prior to breakfast consumption is unknown. However, three previous investigations may aid our understanding of the impact of regular breakfast consumption when endurance exercise sessions are undertaken in the afternoon in the fed state [68,70].

An investigation by Terada and colleagues investigated the impact of performing thrice-weekly sprint interval training either fasted prior to the morning meal or following a high carbohydrate breakfast along with supplemental carbohydrate intake during the exercise session over the course of 4 weeks in 20 male cyclists (cycle > 3 h per week) [120]. Throughout the length of the investigation participants performed 4–7 bouts (week 1: four, week 2: five, etc.) of 30 s all-out cycling efforts against 7.5% of their body mass separated by 4 min of unloaded cycling. Those in the breakfast condition were instructed to consume at least 2.5 g/kg of body mass 1 h prior to the morning exercise sessions (exact intake was not reported) and then consumed a 591 mL bottle of gatorade providing 35 g of carbohydrate throughout the exercise session. Those in the fasted training group were required to fast for at least 10 h prior to arriving and only consumed water during the workout. Following the 4-week investigation, peak power output was lower in the fasted than the carbohydrate group (3664.9 vs. 3871.7 Joules/kg). However, no differences were found for improvements in VO_2_max. Total daily energy intake was not reported, so it is unknown how that may have influenced these results.

Contrary to these findings, in 2020, researchers aimed to further investigate the long-term (4 weeks) effects of omitting the morning meal in a group of 16 elite (three competitive seasons on an elite team) male cyclists [68]. Participants were divided into either a TRE group or a normal-diet (ND) group, which consumed their daily meals at 10 a.m., 1 p.m., and 6 p.m., and 7 a.m., 12 p.m., and 7 p.m., respectively. Both groups consumed an additional snack either before or after the daily training session, although the direct timing of the snack to the workout was not reported. All training was completed in the afternoon during the daily eating window and the cyclists performed ~500 km/week of moderate steady-state cycling throughout the investigation. After the 4 weeks, no significant differences were noted for changes in VO_2_peak (TRE: 4.83 to 4.59 L of O_2_/min; ND: 4.90 to 4.85 L of O_2_/min) or absolute peak power output (TRE: 407.5 to 415 watts; ND: 411.25 to 403.75 watts). However, a significant interaction for relative peak power output was found due to the TRE group increasing this ratio by 4% and the ND group decreasing by 2% (TRE: 6.08 to 6.31 watt/kg; ND: 5.71 to 5.6 watt/kg). Although, neither post hoc pairwise comparison reached statistical significance (TRE: *p* = 0.06; ND *p* = 0.44), and this change was driven by the decrease in body mass only observed in the TRE group (TRE: 67 to 65.8 kg; ND: 72.3 to 72.5 kg). Furthermore, no alterations were seen in any performance metric throughout the endurance test comprised of cycling for 45 min at 45% of their peak power output. These divergent findings in cyclists may stem from either level of the athlete (elite vs. recreationally trained), total energy intake, or differences in training modality (interval vs. periodized continuous training). More investigations are needed to truly ascertain the impact of breakfast on cycling performance.

A subsequent 2021 investigation by Brady and colleagues aimed to investigate if similar effects would be found on running performance in a group of 23 trained (≥5 days a week for at ≥2 years) male medium- and long-distance (>1500 m) runners [70]. Those in the TRE group (n = 12) were required to restrict all daily food intake to an 8 h window, while the normal-diet group (n = 11) participants were solely asked to maintain their normal daily eating habits. Furthermore, all daily food intake was uncontrolled, where participants were only instructed on when to eat, not what to eat. After the 8 weeks of participants following their habitual training routine, once again the researchers failed to note any differences in indices of endurance performance (e.g., VO_2_peak, lactate threshold, running economy, etc.), despite the TRE group self-reporting consuming 265–394 kcals less per day than the ND group leading to TRE-specific reductions in body mass, fat mass, and fat-free mass. These data taken together suggest that breakfast consumption may not significantly impact endurance training adaptations when training is still performed within the fed state. However, more investigations examining the impact of regular fasted exercise and those not directly employing TRE are needed to confirm or refute these hypotheses.

## 5. Conclusions

These collective data suggest that the role breakfast consumption or omission may play for an athlete is highly contextual and there is no one-size-fits-all approach. Depending on the context, consuming breakfast was found to either benefit, impede, or have no impact on the selected outcome. For instance, breakfast consumption may be more impactful with respect to acute performance than longitudinal adaptations to exercise or changes in body composition. Consuming breakfast consistently improved morning endurance exercise lasting >60 min, regardless of intensity, and afternoon/evening aerobic time trial performance. This impact in time trial performance was found in both energy-controlled [41] and -uncontrolled conditions [39,40], suggesting that breakfast may benefit endurance athletes on competition or high-volume training days. However, many of the documented decrements in performance following breakfast omission may, in part, stem from psychological biases [42,45], as the majority of investigations were conducted in those who regularly consume breakfast, and performance decrements were ameliorated when a placebo breakfast was provided. Additionally, performing low-intensity “recovery” sessions fasted prior to consuming breakfast has previously been found to improve mitochondrial density and metabolic adaptations to endurance exercise [116,117]. However, the literature regarding whether these improvements result in better performance is mixed [118,119].

The morning meal’s impact on acute resistance training and resistance training-related adaptations appears to be minimal. The consumption or omission of breakfast did not appear to impact longitudinal improvements in muscular performance over the course of 4–8 weeks in TRE settings or acute afternoon heavy multijoint resistance training (>80%) performed later in the afternoon when total pre-exercise food consumption is consistent. Conversely, reduced early-set (e.g., sets 1 and 2 out of four), but not later-set, repetition performance was seen. Yet, again, this was ameliorated when a placebo condition was included, suggesting more psychological than physiological reasons for alterations in performance.

Lastly, omitting breakfast improved reductions in body mass and body fat over the course of 4–8 weeks in ad libitum non-kilocalorie-controlled conditions. This was primarily caused by an unconscious spontaneous reduction in total daily energy intake. As such, similar benefits were not seen in isocaloric conditions. Thus, skipping breakfast may benefit those looking to reduce body mass in non-kilocalorie-controlled conditions. However, as the majority of the longitudinal literature comes from those practicing TRE, direct investigations solely examining the impact of breakfast outside the scope of TRE are needed. Future investigations should seek to explore the impact of breakfast consumption using more varied exercise protocols (e.g., HIFT, HIIT, high-volume or high-repetition resistance training), populations (i.e., females, older adults, highly trained populations, etc.), longer time periods (>8 weeks), and the degree to which the composition of breakfast may matter to these results. The decision whether to consume or omit breakfast should be based on the individual’s dietary needs along with the goal of the exercise session and program as a whole. Additionally, considerations should be made, if breakfast is not consumed, that daily macro- and micronutrient needs are still met to avoid inadvertent long-term reductions in health status.

## Abbreviations

The following abbreviations are used in this manuscript: IF: intermittent fasting; TRE: time-restricted eating; BMI: body mass index.
